# Accuracy of dynamic navigation compared to static surgical guide for dental implant placement

**DOI:** 10.1186/s40729-020-00272-0

**Published:** 2020-11-24

**Authors:** Dong Wu, Lin Zhou, Jin Yang, Bao Zhang, Yanjun Lin, Jiang Chen, Wenxiu Huang, Yonghui Chen

**Affiliations:** 1 Research Center of Dental and Craniofacial Implants, Fujian Medical University, Fujian, 350001 China; 2 Department of Oral Implantology, Affiliated Stomatological Hospital of Fujian Medical University, Fujian, 350001 China; 3 Fujian Provincial Engineering Research Center of Oral Biomaterial, Fujian Medical University, Fujian, 350001 China; 4 MDS, Stomatological Key lab of Fujian College and University, Fujian Medical University, Fujian, 350001 China; 5 Department of Stomatology, Zhangzhou Affiliated Hospital of Fujian Medical University, Fujian, 363000 China

**Keywords:** Dynamic navigation, Static surgical guide, Accuracy, Dental implants, Surgeon experience, Implant site

## Abstract

**Objectives:**

To compare the accuracy of dynamic navigation (DN) with a static surgical guide (SSG) for dental implant placement and the influence factors such as the experience of the surgeon and the implant sites.

**Methods and materials:**

A total of 38 implants, which underwent the dynamic navigation**,** and 57 implants which underwent a static surgical guide were enrolled in the retrospective study. Coronal deviation, apical deviation, and angular deviation were compared between the DN and SSG groups, along with the different experience level of surgeons and implant sites in the DN group.

**Results:**

There were no statistically significant differences between the DN and SSG groups, and the experience level of the surgeons and implant sites in the DN group. However, the apical deviation of the DN was slightly higher than the SSG group in the anterior teeth (*P* = 0.028), and the angular deviation of DN was smaller than the SSG group in the molar.

**Conclusion:**

Dynamic navigation can achieve accurate implant placement as well as the static surgical guide. Additionally, the experience level of the surgeon and implant site do not influence the accuracy of dynamic navigation, while the accuracy of DN seems higher than the SSG in molar.

## Introduction

As a method of replacing missing teeth, dental implants have been widely used in clinic. However, with the development of implant technology, there are also various postoperative complications, such as damage to the adjacent structure, aesthetic problems, peri-implant inflammation, and even implant failure [1]. Research has shown that many complications were associated with inaccurate implant positioning [2], while correct implant positioning has obvious advantages, such as good esthetics results, long-term health of soft and hard tissue, and it can ensure optimal occlusion and implant loading [3–5]. In addition, ideal implant location optimizes the design of the final restorations and enables the design and manufacture of a screw-retained restoration, which can avoid the retainment of the adhesion agents [6].

Computer-aided implant surgery (CAIS), based on image data navigation, was introduced into the field of dental surgery to improve the accuracy of implant placement and avoid potential complication in order to meet the criterion of clinical needs [7]. CAIS contains a static surgical guide template and dynamic navigation. Static surgical guide template refers to the location of a virtual implant designed according to the CT data, so as to accurately guide the preparation and placement of the implant under the guidance of a surgical guide template, but the guide template is not allowed to modify the implant position during the operation [8–10]. Dynamic navigation refers to the use of a surgical navigation system for implant placement, which can design of the location of a virtual implant according to the CT data. In addition, the system allows real-time tracking of the implant drills and the patient throughout the operation based on motion tracking technology. The designed implant location and the size, length, width, and shape of the implant can be changed during the operation when it is needed to be changed according to the actual intraoral condition of the patients [10, 11]. Compared to the static surgical guide template, dynamic navigation has the following advantages [12, 13]: (1) the cone-beam computed tomography (CBCT), preoperative design, and operation can be completed in the same day, which greatly reduces time and cost to the patients; (2) reduces the psychological and ergonomic pressure of doctors during the operation; (3) increases safety and predictability; (4) allows for the viewing and modification of the preoperative plan in real-time during the operation; (5) is conducive to cooling, reducing the risk of bone injury caused by heat production; and (6) works when there is insufficient space.

With the emergence of a new generation of navigation systems, it is very important to evaluate their availability and accuracy in clinical practice [14]. However, clinical studies comparing the accuracy of a static surgical guide with dynamic navigation are limited, and most of them are in vitro studies [7]. In this study, we compared the coronal deviation, apical deviation, and angular deviation of dynamic navigation with the static surgical guide in patients with dentition defects, so as to analyze the accuracy of dynamic navigation system in the clinical application of oral implants, and factors like the experience level of the surgeon and implant sites, which may affect accuracy, were analyzed at the same time.

## Materials and methods

### Patient selection

This was a retrospective study of patients with missing teeth and a need for implant placement. From January 2018 to October 2019, data from 95 implants in 54 patients (22 women and 32 men), with a mean age of 37 ± 16.7 years (range 19 to 67 years), were collected from the Department of Oral and Maxillofacial Implant Research Center, Affiliated Stomatological Hospital of Fujian Medical University. All patients were informed about the surgical and restoration treatment procedure. The study design was performed in accordance with the Helsinki Declaration (revised in 2008).

The inclusion criteria were as follows: (1) patients with a defect of dentition who accepted the CAIS protocol, (2) no uncontrolled systemic disease unsuitable for implantation, and (3) good systemic and oral health. The exclusion criteria were as follows: (1) heavy smoker (> 10 cigarettes/day), (2) limited mouth opening, (3) a history of radiotherapy in the head or neck region, and (4) possessing a systemic disease, such as uncontrolled diabetes mellitus, coagulation disorders, and alcohol or drug abuse, making the patient unsuitable for implantation.

There were three surgeons involved in the study, surgeons A and B with experiences of over 15 years were defined as rich experience, while the surgeon C with experiences of only 1 year was defined as poor experience. And all of the surgeons had received systematic training on implant navigation system before surgery.

### Preoperative preparation

All patients were subjected to a general oral examination and cone-beam computed tomography (CBCT) examination by NewTom GiANO (NewTom, Italy) with a voxel size of 0.150 mm, tube voltage of 90 kV, current of 7.00 mA, and exposure time of 9 s. A registration device was fixed onto the operating arch with the silicone elastomer when undertaking the scan procedure in the dynamic navigation group. The registration device was stored in povidone iodine solution for the registration procedure at the time of surgery.

Preoperation implant position designs were performed by one operator who had good skill in the digital implant design for both of the groups. Data from the CBCT was exported in a DICOM file and then imported into the dental implant navigation system software for the dynamic navigation group or 3shape software for the static surgery guide group. Optimal three dimensional implant positioning was planned based on the restorative and biologic, as described by Buser in 2004 [15]. For the static surgery guide group, the teeth-supported guide template was produced in the dental laboratory using a 3D printer (AccuFab-D1, SHINING 3D, China).

All of the patients were given a mouth rinse with 0.2% chlorhexidine 3 days before surgery. A plaster cast was made in the static surgery guide group and send to the dental laboratory.

### Surgical process of dynamic navigation

**Fig. 1:**
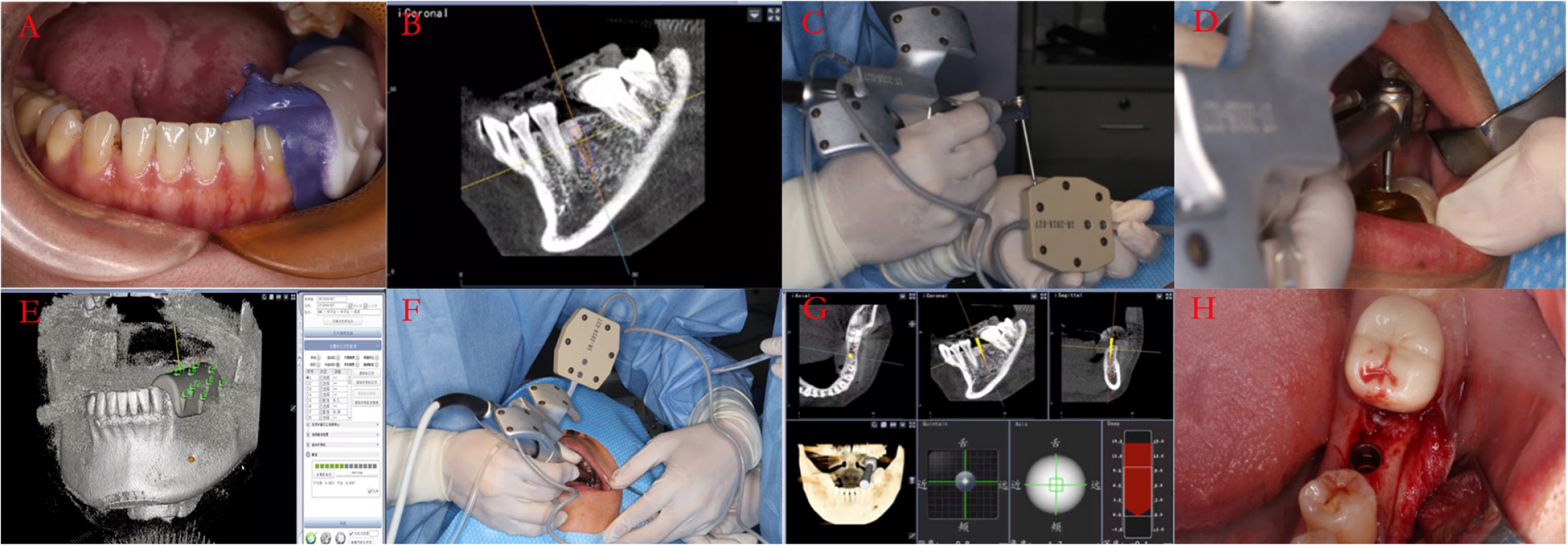
The process of dynamic navigation. **a** The registration device was fixed onto the operating arch with a silicone elastomer when taking the CBCT scanning. **b** The optimal 3D implant position was planned in the dental implant navigation system software. **c** Matching the reference device. **d** Matching the registration plate with the handpiece. **e** The view of matching the registration plate in the software. **f** The process of drilling under navigation. **g** The view of real-time tracking under navigation in the software. **h** Oral image of implant placement

The reference device was fixed onto the teeth according to the manufacturer’s instructions. Then, the tracker was matched with the reference device to position the handpiece. After that, the reference device was fixed onto the implant site using the handpiece, with a drill that matched the registration plate with the handpiece. A full-thickness flap was made under local anesthesia with Primacaine® (4% Articaine, 1/100,000 adrenaline, ACTEON) after the matching process. Then, the drill was used according the implant system we selected under the guidance of the dynamic navigation system (Dental Implant Navigation System, Model: DHC-DI3E, Suzhou Digital-health care Co. Ltd, China). Briefly, the process is shown in Fig. 1.

### Surgical process of static surgical guide

**Fig. 2:**
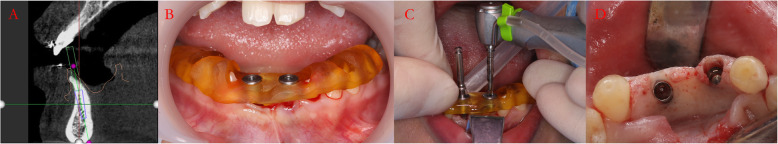
The process of static surgical guidance. **a** The design of the implant optimal position. **b** The surgical guide template was inserted and controlled for stability of the implant position. **c** Using the drill to preparation. **d** Oral image of implant placement

A full-thickness flap was made under local anesthesia with Primacaine® (4% Articaine, 1/100,000 adrenaline, ACTEON). Then, the surgical guide template was inserted and controlled for stability of the implant position. Once stability was confirmed, the implant placement was performed according to the manufacturer’s instructions. Briefly, the process is shown in Fig. 2.

### Postoperative treatment

All patients underwent a CBCT scan with the same settings as previously described after surgery, were given antibiotics for 3 days, and received mouth rinsing for 1 week. The sutures were removed after 7–10 days.

### Accuracy evaluation

**Fig. 3:**
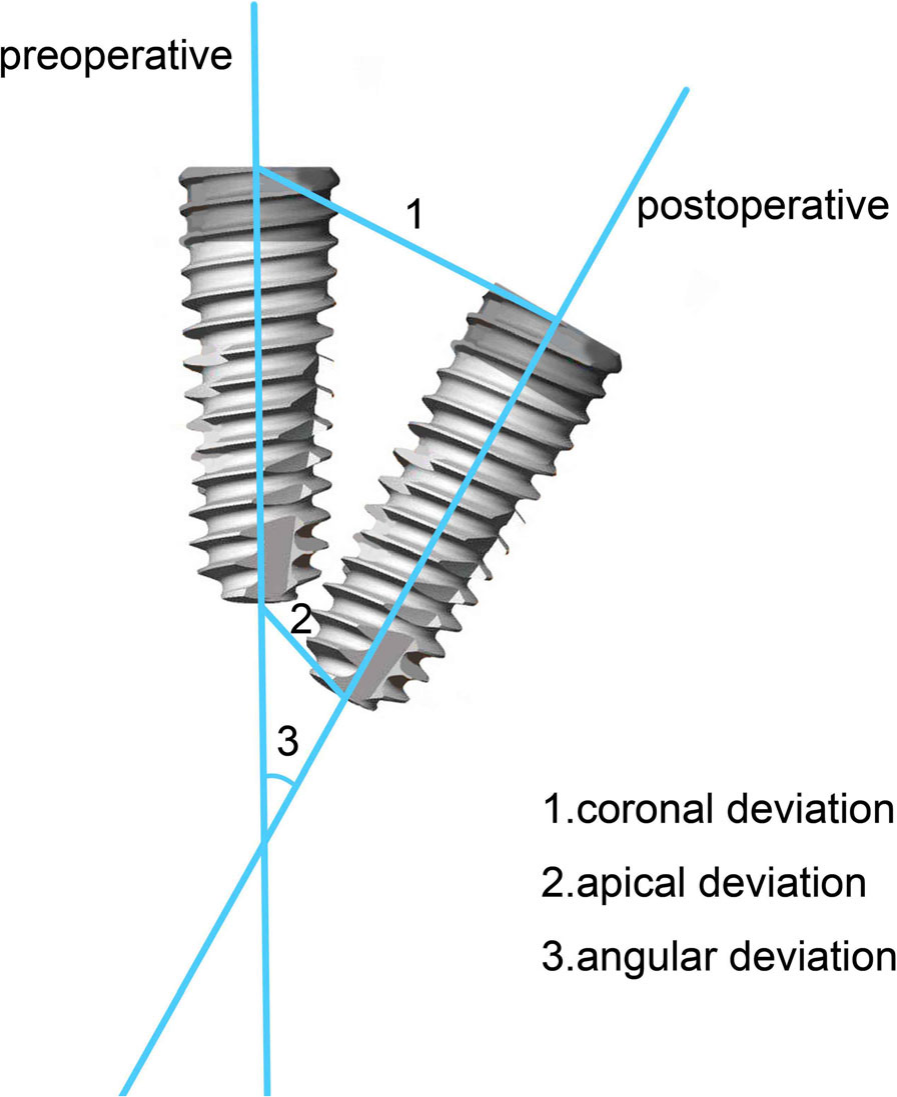
The schematic diagram of measurement accuracy

The preoperative design data of the implant in the dynamic navigation system was exported in the form of a DK file, and the postoperative CBCT image data was exported in the form of a DICOM file. The actual deviation of the implant position could be measured by matching the preoperative and postoperative imaging data in the software. The precision analysis of the static surgical guide was carried out in the similar manner as the dynamic navigation. We analyzed coronal deviation, apical deviation, and angular deviation of the postoperative implant positions with the preoperative designs as they are shown in Fig. 3. The coronal deviation was measured as the distance between the centers of the implant coronal platform. Apical deviation was measured as the distance between the centers of the implant apical. Angular deviation was measured as the angle of the axis of the implant center.

### Statistical analysis

Statistical analysis was carried out using SPSS 22.0 software. The coronal, apical, and angular deviations of the two groups were compared totally and at different positions using an independent-samples *t* test. The variable of surgical experience and the implant sites in the dynamic navigation group were compared using one-way ANOVA, followed by a Scheffe post hoc test. *P* values < 0.05 were defined as statistically significant.

## Results

### Basic information of the implants

In the present study, 38 implants were collected from 25 patients in the dynamic navigation group and 57 implants were collected from 29 patients in the static surgical guide group. The implant locations and the basic information of the patients are detailed in Table 1.

**Table 1:** The basic information of the implants

	Dynamic navigation			Static guide template		
Implant site	Anterior teeth	Premolar	Molar	Anterior teeth	Premolar	Molar
Quantity	11	8	19	21	14	22
Surgeon	Surgeon A	Surgeon B	Surgeon C	Surgeon A	Surgeon B	Surgeon C
Quantity	15	13	10	27	30	0

### Complications

The design position was adjusted intraoperatively in four cases from the dynamic navigation group. The static surgical guide template could not put in the right intraoral position, because of the inaccuracy of the surgical guide in two cases. In the whole study, all of the patients only experienced routine postoperative reactions, such as swelling and mild pain, and no serious postoperative complications (such as postoperative hemorrhage in the floor of the mouth and lower lip numbness).

### Comparison of accuracy

**Fig. 4:**
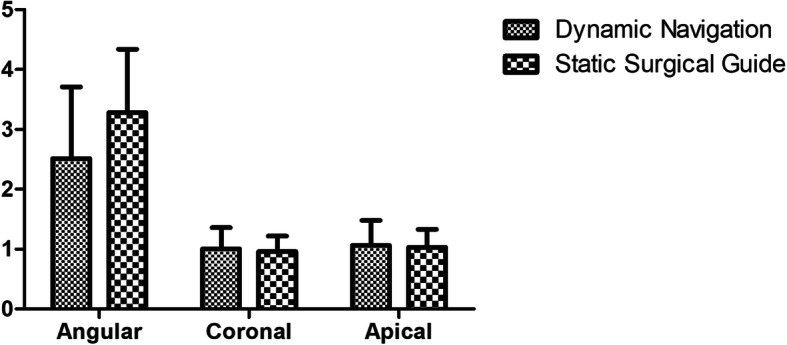
The coronal deviation, apical deviation, and angular deviation of the dynamic navigation and static surgical guide

The coronal deviation, apical deviation, and angular deviation of the dynamic navigation group were (1.36 ± 0.65) mm, (1.48 ± 0.65) mm, and (3.71 ± 1.32)°, respectively, and (1.22 ± 0.70) mm, (1.33 ± 0.73) mm, and (4.34 ± 2.22)° in the static surgical guide group. There were no statistically significant differences between these two groups as shown in Fig. 4.

### Comparison of surgeons

**Fig. 5:**
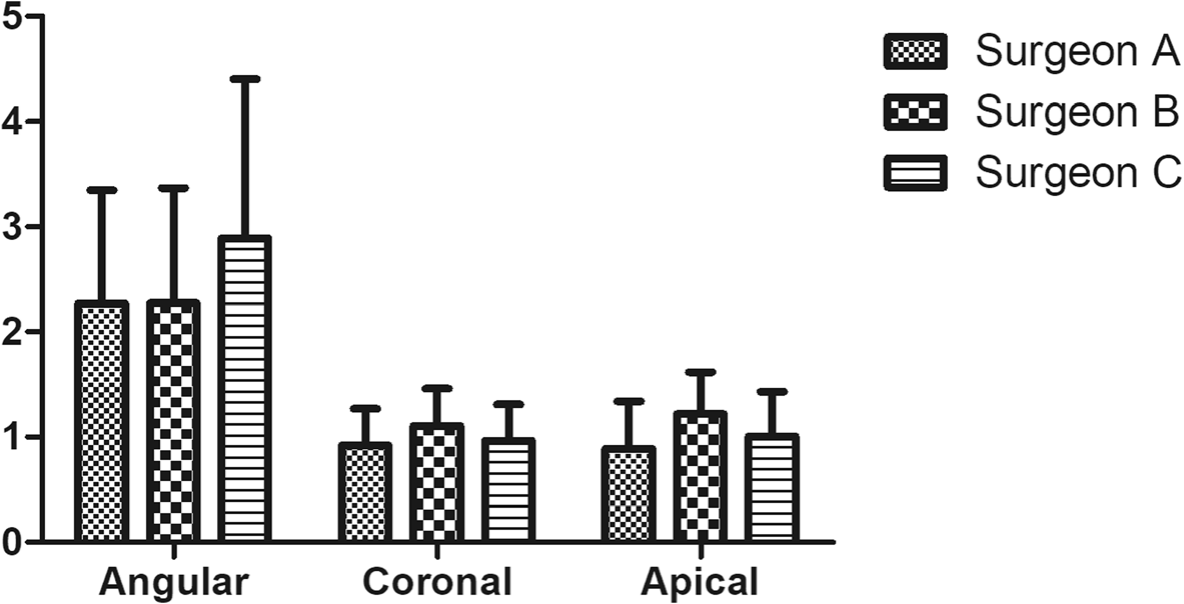
The coronal deviation, apical deviation, and angular deviation of different surgeons in the dynamic navigation group

In terms of the experience of the surgeons, we found no statistical differences between these three different surgeons. There were no differences between the two rich experiences surgeons, or the poor experience surgeon with the rich experience surgeons, as shown in Fig. 5.

### Comparison of implant site

**Fig. 6:**
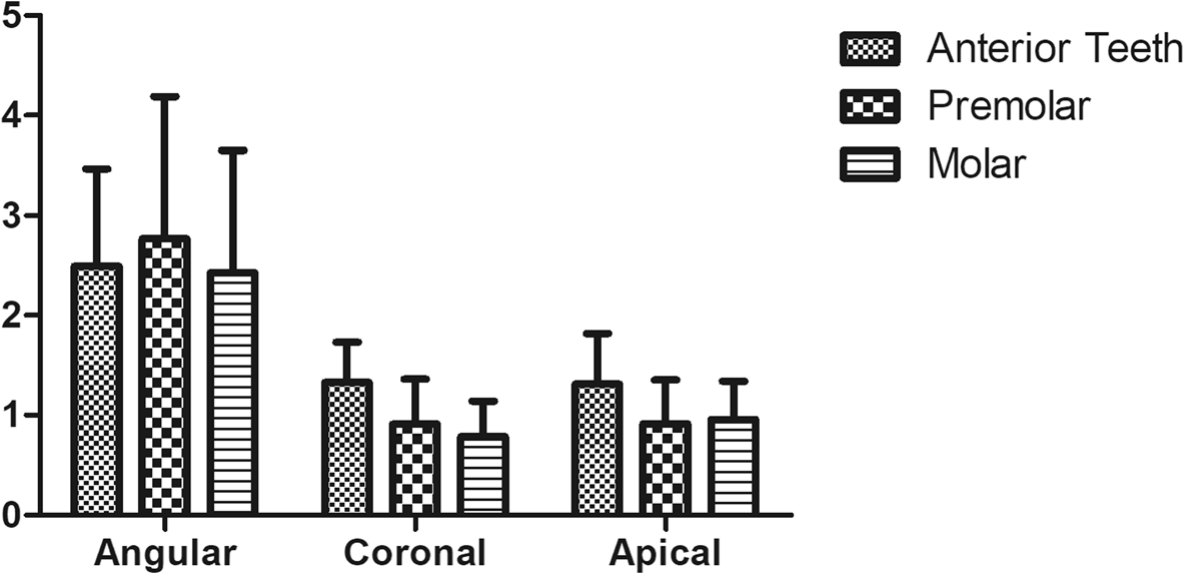
The coronal deviation, apical deviation, and angular deviation of different implant sites in the dynamic navigation group

**Fig. 7:**
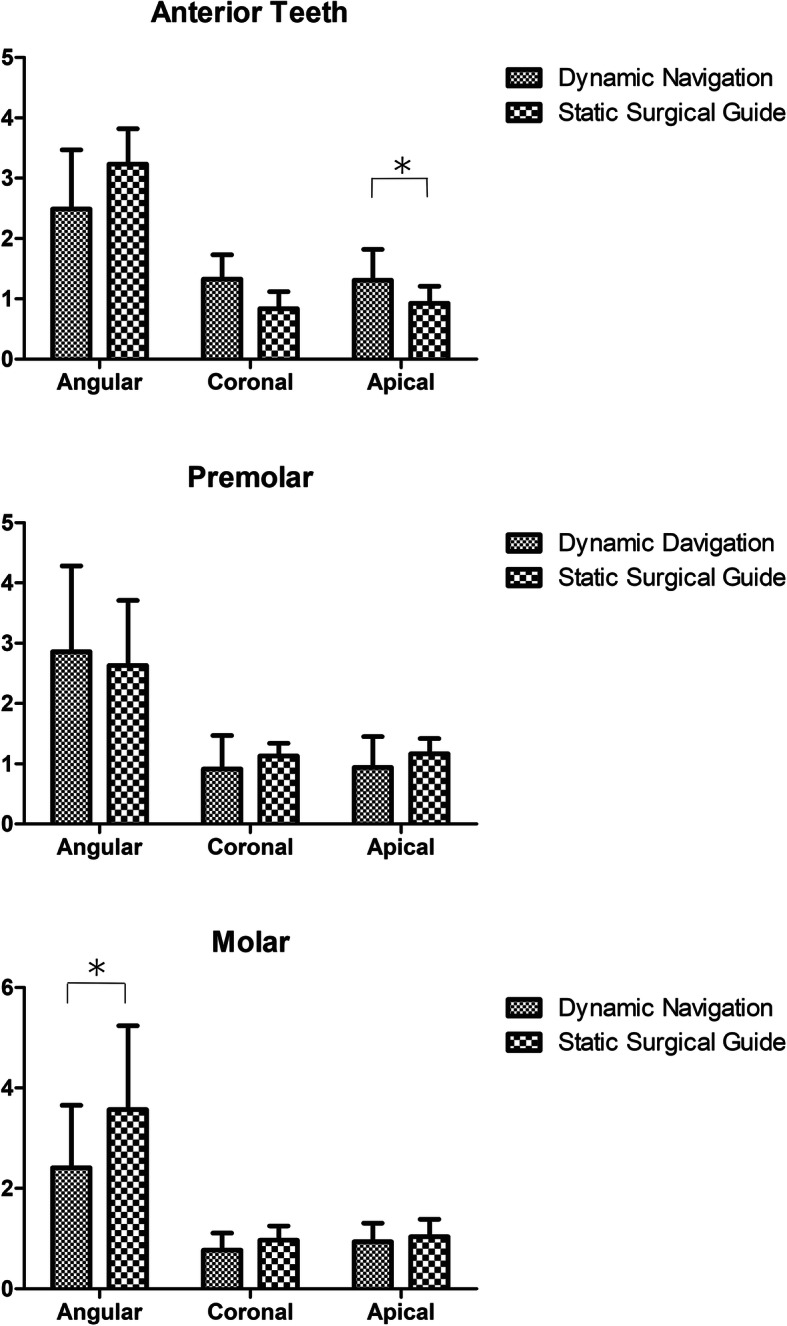
Comparison of the coronal deviation, apical deviation, and angular deviation of the dynamic navigation with the static surgical guide in different implant sites. *P < 0.05, **P < 0.01

We decided the implant sites of the anterior teeth, premolar, and molar in order to further identify whether the implant site may influence the accuracy. There were no statistical differences between each site in the dynamic navigation group as shown in Fig. 6. In addition, we compared the dynamic navigation group with the static surgical guide group at the same implant sites, and the results are shown in Fig. 7. There were no statistical differences between the two groups in the premolar. However, the apical deviation of the dynamic navigation group was slightly higher than in the static surgical guide group in the anterior teeth (*P* = 0.028), and the angular deviation of the dynamic navigation group was smaller than the static surgical guide group in the molar.

## Discussion

The right implant 3-dimensional position is significant to the outcome of final restoration, especially in the esthetic zone and in the mandibular to avoid any damage to the inferior alveolar nerve [16, 17]. In the present retrospective study, dynamic navigation was successfully used in implant placement, and the accuracy was similar to the static surgical guide.

In this study, the results showed that there were no significant differences between dynamic navigation and the static surgical guide in terms of coronal deviation, apical deviation, and angular deviation. The mean value of coronal deviation and apical deviation were less than 1.36 mm and 1.48 mm, and the mean value of the angular deviation was within 4.34°, showing a high accuracy. Some systematic reviews and meta-analyses on the accuracy of the navigation and surgical guide templates showed that the mean coronal and apical deviations of CAIS were less than 1.22 mm and 1.45 mm, respectively, and the angular deviation was less than 4.06° [10, 18, 19]. Guzmán et al. compared the deviation of dynamic navigation and three commercial static surgical guides in the preparation of implants in an in vitro model. The results showed that the mean deviation of the coronal, apical, and angular were less than 0.85 mm, 1.20 mm, and 4.00°, respectively [7].

The reason why these results were a little lower than ours may be that they were all in vitro studies. Compared with an in vitro study, the possible factors which may affect accuracy are relatively hard to handle in a real clinical situation. These factors are gingiva, mucosa, blood, saliva, and the degree of mouth opening. Some research has found that apical and angular deviations were 1.56 mm and 3.62° in in vivo measurements, which were almost quadruple those of the in vitro one, when using the same dynamic navigation system [13, 20]. However, an in vitro study can better explain the accuracy of the dynamic navigation itself, while an in vivo study and practical clinical application can give us more of a clinical guide.

When we analyzed the different surgeons in the dynamic navigation group, we found that there was no statistical difference in the influence of different experience levels of the surgeons on accuracy. Sun et al. proved that the implant navigation system could help a dentist to insert the implant into the correct position accurately, without the influence of the operational experience [21]. Rungcharassaeng et al. compared experienced with inexperienced surgeons in the accuracy of dental supported static surgical guide templates, and the results showed no significant difference [22]. In the present study, all of the surgeons had received systematic training on implant navigation system before surgery, and there was no statistical difference between the experienced and inexperienced surgeons, also between the two rich experienced surgeons. Therefore, we demonstrated that surgical experience in dental implantation would not influence the accuracy of dynamic navigation.

We derived from Fig. 6 that the implant site would not influence the accuracy of dynamic navigation. This is consistent with the results of Stefanelli et al., who analyzed the implant site in a retrospective study of dynamic navigation, and the results showed that the implant site had no significant influence on accuracy [23]. Nevertheless, Block pointed out in their study that the deviation in the posterior tooth area was larger than that in the anterior tooth area [24]. We found that the angular deviation of the surgical guide was larger than that of dynamic navigation in the molar. This may be related to the patient’s open-mouth degree, because the surgery guide template has a certain thickness, the final angle may be offset due to the open-mouth degree during implant placement in the molar area.

The accuracy of implant placement by using CAIS may be affected by various aspects, such as image acquisition, image data processing, surgical guide plate production, support type of surgical guide template, preparation, implant placement, registration process, and human error [25–27]. Some factors may affect the accuracy of dynamic navigation and surgical guide simultaneously, such as CBCT image quality, bone density, etc. Since the design of the implant surgical plan is based on the CBCT images in the navigation system or surgical guide design software, any deformation or error in the CBCT images could lead to incorrect planning, which will affect the accuracy of the final implant position [28, 29].

Compared with the static surgical guide, dynamic navigation has many advantages, for example, it can be completed in full, from the preoperative design to the surgical completion, in the same day, which greatly saves the patient’s time and cost. However, the current dynamic navigation equipment is expensive, and its accuracy still needs to be verified by more clinical studies. In addition, the surgical guide can achieve satisfactory results with a lower price for both doctors and patients, while dynamic navigation requires a period of additional training and learning before surgery.

### Limitations of the study

There was selection bias in the present study as a retrospective study. The sample size was not large enough. A prospective randomized study is needed to measure the accuracy of dynamic navigation and its influencing factors.

## Conclusions

The accuracy of dynamic navigation is similar to that of a static surgical guide, which can meet the clinical needs of daily oral implant placements. Meanwhile, the planting sites had no obvious influence on the accuracy results. The apical deviation of the dynamic navigation was slightly higher than the static surgical guide in the anterior teeth, and angular deviation of dynamic navigation was smaller than the static surgical guide template in the molar. And the experience of a surgeon may not influence the accuracy of dynamic navigation once he got good training about it.

## Data Availability

The datasets used and/or analyzed during the current study are available from the corresponding author on reasonable request.
